# High-resolution climate–dengue modeling and mid-century projections under SSP5-8.5 in Costa Rica

**DOI:** 10.1016/j.soh.2026.100157

**Published:** 2026-04-27

**Authors:** Hugo G. Hidalgo, Eric J. Alfaro, Fabio Sanchez, Adriana Troyo, Tito Maldonado, Zaray Miranda-Chacón, Eric Morales-Mora, Monserrat Solano-Gamboa, Marco Acosta-Quesada

**Affiliations:** a School of Physics, Center for Geophysical Research and Center for Research in Pure and Applied Science, University of Costa Rica, San Pedro, San José, 11501-2060, Costa Rica; b Center for Geophysical Research, Center for Research in Marine Sciences and Limnology and Center for Research in Pure and Applied Science, University of Costa Rica, San Pedro, San José, 11501-2060, Costa Rica; c Center for Research in Pure and Applied Mathematics, University of Costa Rica, San Pedro, San José, 11501-2060, Costa Rica; d Vectors Research Laboratory, Center for Research in Tropical Diseases, Faculty of Microbiology, University of Costa Rica, San Pedro, San José, 11501-2060, Costa Rica; e School of Physics and Center for Geophysical Research, University of Costa Rica, San Pedro, San José, 11501-2060, Costa Rica; f School of Medicine, University of Costa Rica, San Pedro, San José, 11501-2060, Costa Rica; g School of Health Technologies, Department of Environmental Health, University of Costa Rica, San Pedro, San José, 11501-2060, Costa Rica; h Center for Geophysical Research, University of Costa Rica, San Pedro, San José, 11501-2060, Costa Rica

**Keywords:** Dengue, Climate change, Meteorology, Central America, Variability, Parsimonious

## Abstract

**Background:**

Climate change may expand dengue transmission in space and season across Central America. In Costa Rica, complex topography and very small districts mean coarse global climate models can miss local conditions that drive outbreaks, creating a need for district-level, high-resolution climate–dengue assessments. This study aims to: (1) model the climate–dengue relationship at the district level using high-resolution data; (2) identify the best climate predictors for dengue incidence; and (3) provide mid-century (2035–2065) dengue cases projections under a pessimistic scenario (SSP5-8.5) with seasonal windows actionable by region.

**Methods:**

Precipitation and temperature indices derived from the Climate Hazards group Infrared Precipitation with Stations (CHIRPs) and Climate Hazards Center Infrared Temperature with Stations (CHIRTs) were related to dengue diagnoses from Costa Rica's public health centers using a linear model. An objective algorithm selected parsimonious climate–dengue predictors, with cross-validation to prevent overfitting. The resulting quasi-optimal models combined with downscaled projections from an ensemble of eight General Circulation Models (GCMs) to estimate future dengue incidence changes at the district level, Costa Rica's smallest administrative division.

**Results:**

Temperature and precipitation data are significantly related to dengue counts. Temperature dominates most district models during the dry season (December to June), while precipitation dominates during the rainy season (July–October). Mid-century projections indicate increases of up to 42 additional cases in some districts compared to the historical baseline, with the location of the most pronounced changes varying by month.

**Conclusions:**

The projected dengue increases presented here are driven solely by climate change under the most pessimistic greenhouse gas (GHG) concentration scenario, and thus represent a potential upper bound on future risk. These findings offer actionable guidance on where and when dengue incidence may rise, and should inform adaptive health policies aimed at reducing the impacts of climate change in high-risk areas.

## Highlights

• A procedure is developed for determining which precipitation- and temperature-derived indices, as covariates, and at what lags are associated with total dengue cases across most districts in Costa Rica.
• Parsimonious quasi-optimal regression models are identified for each district, ensuring low collinearity among covariates.
• Temperature-related covariates are prevalent across many districts during the dry season (November–June), while precipitation-related covariates are more commonly associated during the rainy season (July–October).
• Using simulations from General Circulation Models as input, the quasi-optimal regression models are applied to project mid-century dengue incidence under a pessimistic greenhouse gas emission scenario (SSP5-8.5).
• In the most extreme cases, a few districts project mid-century (2035–2065) increases of approximately 42 cases compared to the historical baseline period (1985–2015).

## 1 Introduction

The limited success of international efforts to reduce global warming to levels established in the Paris Agreement, together with the increasing frequency and intensity of climate impacts, underscores the urgency of adaptation in Central America and the wider Latin America and Caribbean region [1]. Recent syntheses indicate high confidence that climate change will increase the risk of severe health impacts in Central America, including larger and more frequent vector-borne disease epidemics such as dengue, by the end of the 21st century [2,3]. However, adaptation decisions in public health are made locally, and the evidence base remains disproportionately coarse: regional and national assessments rarely resolve the subnational heterogeneity needed to target surveillance, vector control, and preparedness [1,3]. This study, therefore, focuses on dengue-specific projections at the district scale in Costa Rica, aligning projected risk with the spatial unit at which dengue is reported and where operational decisions are implemented.

Dengue is widely considered a climate-sensitive disease because fluctuations in temperature, humidity, and rainfall affect mosquito biology and behavior, including development rates, survival, biting frequency, and ultimately transmission potential [4,5]. At the population level, temperature effects on dengue incidence are consistently nonlinear and moderated by local climatic and socioeconomic context, while extremes such as heatwaves can further increase risk [6,7]. Precipitation plays an important, often lagged and context-dependent role by creating or removing larval habitats, with systematic evidence showing robust associations between rainfall and dengue at monthly time scales [8]. These climate-driven effects are further shaped by non-climatic drivers such as travel, urbanization, and mobility, which influence introductions and human-vector contact patterns [9,10]. Together, these mechanisms imply that changes in climate can shift not only the average level of risk but also the timing and location of outbreaks—features that matter directly for seasonal preparedness and resource allocation.

Costa Rica is a particular challenge case for district-scale analysis. The country's narrow landmass between two oceans and steep topographic gradients generate sharp climatic contrasts over short distances, producing localized temperature and rainfall regimes that can differ substantially across neighboring areas [3]. At the same time, dengue surveillance and planning operate at the district level, the smallest administrative division in the country, and routine case reports from public health centers are disaggregated at this level. In practical terms, this makes the district the minimum unit at which dengue risk can be consistently linked to climate data and acted upon by local public health operations, especially because some districts cover only a few square kilometers. Consequently, projections at broader spatial scales risk averaging across distinct microclimates and obscuring localized hotspots that drive operational burden.

Despite growing evidence that climate variables combined with statistical or machine-learning methods can provide useful predictive skill for dengue risk in Costa Rica, extending these relationships to climate-change projections remains challenging [11]. A central limitation is the scale mismatch between decision-relevant units (districts) and the coarse spatial resolution of global climate models, which typically cannot represent the local climate variability that governs district-level transmission conditions [12]. A second limitation is that dengue projection studies often rely on predictors or modeling choices that are not readily transferable to future scenarios at fine spatial scales, increasing uncertainty or reducing interpretability for preparedness planning. Recent advances partly resolve the climate-data barrier: high-resolution projections based on downscaled Coupled Model Intercomparison Project Phase 6 (CMIP6) simulations are now available for Central America, providing kilometer-scale temperature and precipitation estimates suitable for complex terrain [13]. In parallel, regional evidence indicates that both natural climate variability and long-term warming can reshape the distribution of *Aedes* vectors and dengue burden, and that limiting warming could reduce the incidence and spatial spread of dengue in Latin America [14,15]. These developments create a timely opportunity to translate high-resolution climate projections into dengue-specific, district-scale information that better aligns with operational public health needs.

Here we address this gap by developing projection-ready dengue-climate models at the district level in Costa Rica and using them to estimate potential mid-century dengue incidence (2035–2065) under a high-emissions scenario (SSP5-8.5). Our analytical contribution was three-fold: (1) we quantified district-level dengue–climate relationships using high-resolution climate data aligned with the surveillance unit; (2) we applied an algorithmic predictor-selection approach to produce parsimonious, cross-validated models designed to reduce overfitting while retaining predictors available in climate projections; and (3) we translated downscaled CMIP6 estimates into actionable seasonal projection windows summarized by Costa Rica's climate regions to support preparedness planning and targeted adaptation.

## 2 Methods

### 2.1 Data

#### 2.1.1 Total dengue cases

Total dengue cases (suspected, probable, and confirmed dengue cases) consisting mostly of clinical reports were obtained by the Ministry of Health from public health centers in Costa Rica during 2011–2023. Dengue is a mandatory notifiable disease in Costa Rica, and all cases must be reported to the Ministry of Health individually. The data consisted originally of individual reports of diagnosed cases from the public health centers of Costa Rica (private cases were not reported but they are usually a minority compared to the medical attention in public clinics and hospitals). There is no denominator in the data, each entry in the database is an individual record of the patient, including the date, location of the district, municipality and province of the clinic or hospital, sex of the patient, race, patient's age (in years, months, and days), nationality, occupation, name of the hospital or clinic, method of diagnostic, Ministry of Health's region name, and other minor ancillary (but usually missing) information in the records. It is unknown if diagnosing practices may affect these records, but these types of improvements are not common in the country. Details of the data can be found at the database of Ministry of Health of Costa Rica [16,17]. Reported dengue cases were aggregated at monthly intervals for each district.

#### 2.1.2 Observed climate data

A gridded blend of satellite and meteorological station precipitation (*P*) data from the Climate Hazards group Infrared Precipitation with Stations (CHIRPS) [18] was used to represent observations from 1981 to 2023. These data have a horizontal spatial resolution of approximately 5 km × 5 km. Similarly, daily maximum and minimum temperature data were obtained from a corresponding dataset [19] for the period 1983–2023. This data set is called Climate Hazards Center Infrared Temperature with Stations (CHIRTS) [19]. The average monthly temperature (*T*_avg_) was computed from the mean of maximum and daily temperatures of the month. Both CHIRPS and CHIRTS datasets have shown good agreement with station data in several locations across Central America and the Caribbean region [[20], [21], [22], [23], [24], [25]]. Alfaro-Cordoba et al. [24] provided detailed comparison statistics in Supplementary Material 2 of their study. The daily precipitation data were used to create four monthly indices: (1) mean monthly precipitation (*P*_mon_), (2) number of rainy days defined as days with *P* ≥ 0.1 mm (days*P*), (3) number of days of each month exceeding the 80th percentile of the 1983–2023 climatological daily precipitation (*P*_80_), and (4) number of days below the 20th percentile (*P*_20_). Table 1 shows the description of the climate variables.

**Table 1:** Description of variables used as covariates.

Variable	Description	Unit
*P*_mon_	Monthly mean precipitation from daily data	mm
days*P*	Number of rainy days from daily data	d
*P*_20_	Number of days of the month with precipitation lower than the 20th percentile precipitation for each month over all years (2011–2023)	d
*P*_80_	Number of days of the month with precipitation higher than the 80th percentile precipitation for each month over all years (2011–2023)	d
*T*_max_	Monthly mean maximum temperature	°C
*T*_min_	Monthly mean minimum temperature	°C
*T*_avg_	Monthly mean average temperature: *T*_avg_ = (*T*_max_ + *T*_min_)/2	°C

Note: All covariates were tested at 0, −1, −2 month lags with respect to total dengue cases.

#### 2.1.3 Model climate projections data

Daily data for the same precipitation and temperature variables as for the CHIRPS and CHIRTS data from general circulation models (GCMs) at 1 km × 1 km spatial resolution for Costa Rica were obtained from our previous study [13]. The daily model data were used to produce the same monthly indices as for the CHIRPS and CHIRTS data. Data were sourced from simulations of eight GCMs (Table 2) from the CMIP6, the most recent iteration of coordinated climate model simulation. The data were downscaled for each model individually, and the ensemble was produced by averaging the results of all different model outputs as done by Hidalgo et al. [13], and details of the method can be found there. The data contains daily simulations of *P*, daily maximum temperature (*T*_max_*),* and daily minimum temperature (*T*_min_) from 1985 to 2065. Average temperature was computed from the average of *T*_max_ and *T*_min_ in the same manner as with the CHIRT data.

**Table 2:** General circulation models (GCMs) used in this study.

Model	Native resolution
ACCESS	1.8750° × 1.2500°
AWI	0.9375° × 0.9375°
CAMS	1.2500° × 1.1215°
EC-EARTH3	0.7031° × 0.7031°
EC-EARTH3-veg	0.7031° × 0.7031°
MPI	0.9375° × 0.9351°
GFDL	0.7031° × 0.7031°
UKESM1	1.8750° × 1.2500°

#### 2.1.4 Climate regions of Costa Rica

A geographical layer of the delimitation of Costa Rica's climate regions was obtained from the National Meteorological Institute of Costa Rica [26]. These data were used for averaging the climate and dengue data to obtain seasonal cycles of those variables by climate regions.

#### 2.1.5 Administrative boundaries

The delimitations of municipalities (local governments) and districts (the administrative units that conform to a municipality) in Costa Rica were obtained from the Geographical National Institute of Costa Rica [,^27^]. The population data from the 2022 census was obtained from National Institute of Statistics and Censuses (INEC in Spanish) [28].

### 2.2 Statistical modeling approach

The seasonal cycles of the dengue total cases by climate region were calculated and compare with the seasonal cycles of precipitation and temperature. This comparison could provide a general assessment of the potential relationships between dengue seasonality and the seasonal availability of water (which promotes vector proliferation) and temperature (which influences vector fitness).

Although the climate data is originally at daily resolution, the climate indices are calculated at monthly time-step from the daily data. We selected the monthly scale because finer temporal aggregations (e.g., weekly data) would produce many zero values in both dengue and precipitation data, which would result in non-Gaussian distributions, and would violate the assumptions of the linear methods employed here. For each district and month, we related the climate indices listed in Table 2 to the dengue total cases. The covariates included lagged versions of these indices at −2, −1, and 0 month. Given the potential for overfitting (3 lags × 7 indices = 21 covariates), we applied an objective section algorithm based on previous research [29,30] to identify parsimonious models with cross-validation. The procedure consists of the following:

(1) Given a set of potential covariates of the models (*X*_1_*, X*_2_*, X*_3_*, … , X*_*n*_) where *n* is the total number of indices with their lags (*n* = 21) for *each* district, and *Y* is the predictand variable (in this case, the average dengue total cases for that district).
(2) Calculate the *r*_*n*_ partial correlations of the potential predictors with the predictand variable *Y.*
(3) Start with models of only one predictor (in a “forward” introduction of variables approach) and consider only for those variables where the partial correlation is statistically significant.
(4) Starting with the predictor with the highest absolute *r*_*n*_, evaluate the leave-one-out cross-validation standard error (CVSE) by deleting one month and using the rest of the data to predict it. Then, the second month is removed, the prediction is stored, and the process repeats. At the end of the series of predictions, the methodology results in a time series $\hat{Y}$, which is compared to *Y*, and the error is computed according to the following formula:

(Eq. 1) $$\mathrm{CVSE}=\sqrt{\frac{\sum_{i}^{m}{\left(Y_{i}-{\hat{y}}_{\left(i\right)}\right)}^{2}}{\left(n-p\right)}}\text{,}$$

where $Y_{i}$ is the value of the predictand (dengue total cases) at month *i*; ${\hat{y}}_{\left(i\right)}$ is the fitted response of the *i*th month with the *i*th observation removed, *m* is the number of months in the dataset, and *p* is the number of regression coefficients.

(5) For all possible 1-variable models, the CVSE was calculated. The model with the minimum CVSE was retained as the candidate solution
(6) Then, the model of the predictor selected as the best for a 1-variable combination will be added one variable at a time (in order of partial correlation) to produce possible models with two variables.
(7) The 2-variable models were selected according to the following sub procedure (see also [30]).
  (a) The retention of variables in the models depends on two tests: a *t*-test and a sign test.
  (b) Principal component analysis (PCA) was performed on the two candidate variables, resulting in two principal components (PC1 and PC2).
  (c) A linear regression of PC1 with *Y* was performed.
  (d) If the regression coefficient of the equation was statistically significant in the *t*-test, then the sign test was subsequently applied.
  (e) The signs of the regression coefficients of *Y* with PC1 were expressed in terms of the original variables by multiplying the PCA regression coefficients by the PCA eigenvectors. These signs were then compared with the partial correlations of the two variables. If the signs were consistent, the sign test was passed. In this case, PC1 was retained and the model was accepted as a viable option.
  (f) Next, PC1 and PC2 in the regression was tested, following the order of variance explanation.
  (g) If PC1 and PC2 did not pass the *t*-test, only PC1 was retained and the procedure was terminated.
  (h) If PC1 and PC2 passed both the *t*-test and the sign test, PC1 and PC2 were retained in the model.
  (i) When more than two variables were evaluated, PC3 was subsequently added to the model according to the following criteria: If using PC1 and PC2 passed the *t*-test but failed the sign test, PC1 and PC2 were temporarily retained and PC3 was added. If PC1, PC2 and PC3 passed both the *t*-test and the sign test, PC1, PC2 and PC3 were retained; otherwise, only PC1 was retained.
  (j) A new subset of variables was then tested, and the procedure returned to step (b).
(8) All possible combinations of two variables were tested. If the CVSE for 2-variable combinations was larger than that for the 1-variable model, the 1-variable model was accepted and the procedure was terminated.
(9) If the 2-variable combinations yielded a lower CVSE, then the combinations of three variables were tested. This process continued until the model with the lowest CVSE (the most parsimonious) was identified.

Seven climate predictor variables (listed in Table 2) were evaluated at three lags (0, −1, −2 months), yielding a total of 21 variables. When selecting *k* variables from these *N* = 21 variables, the number of possible combinations, denoted as C(*N,k*), $\left(\begin{array}{c} N\\ k \end{array}\right)$ or “*N* choose *k*” can be very large. This number is calculated using the combinatorial formula involving factorials [31]:

(Eq. 2) $$\left(\begin{array}{c} N\\ k \end{array}\right)=\frac{N!}{k!\left(N-k\right)!}\text{.}$$

Due to the factorials of possible combinations, the number of combinations quickly becomes very large as *N* increases, make it computationally prohibitive to test all possible combinations for large *N* values. Therefore, only three time lags were tested. Although higher-order time lag relationships between climate and dengue may exist, the search was limited to *N* = 21 to maintain computational feasibility within the available resources. For *N* = 21, a maximum of 5,000,000 combinations was tested for each *k* and each district. Within each subset, the variables were introduced in the order of their decreasing partial correlation strength. This procedure satisfies the requirement of parsimonious models and verifies that the predictor variables represent the proper relationship with the predictand variable and are conceptually acceptable [29,30], thus reducing the probability of overfitting.

**Fig. 1:**
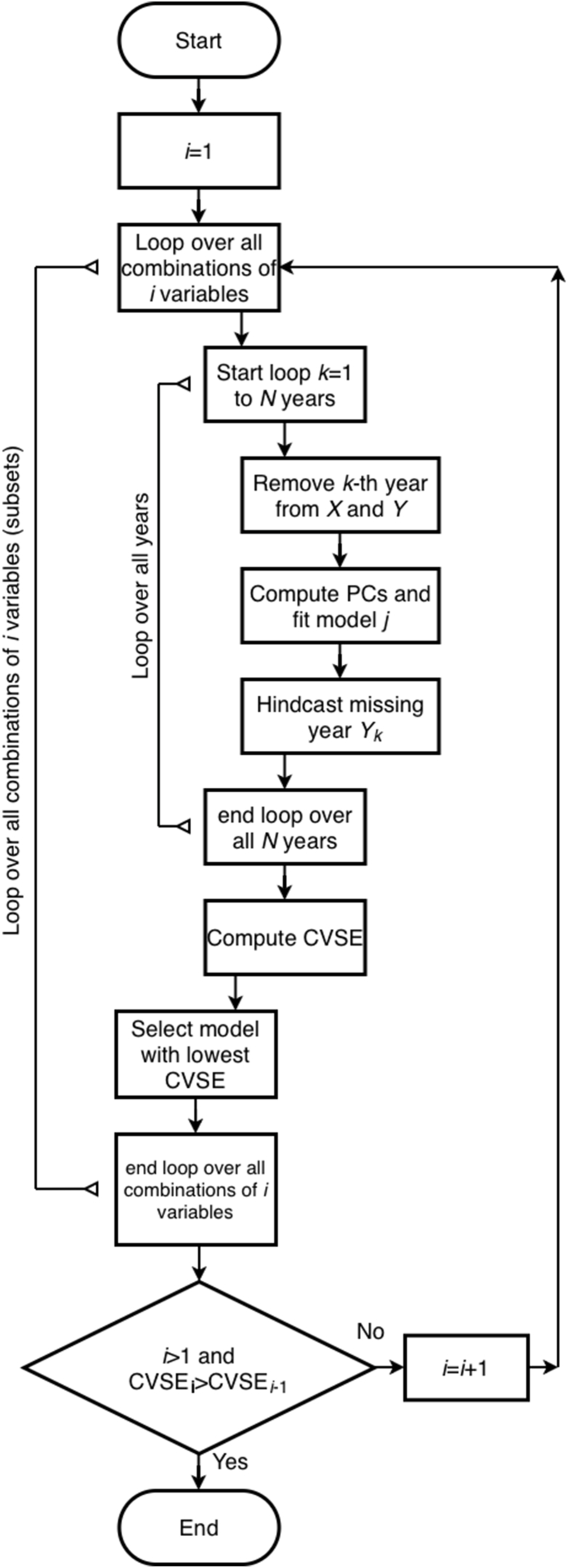
Flow chart of the procedure for selecting variables to identify quasi-optimal parsimonious models in each district. The algorithm used for identification of the optimal model parameters. Given: *X*(1 … *N*, 1 … *p*), predictor variables matrix, where *p* is the maximum number of predictor variables and *N* is the number of years. *Y*: predictand variable matrix, *Y*(1 … *N*, 1). CVSE is cross-validation standard error. Note that the best variable subset combination is the one that minimizes CVSE_*i*_. The combinations of *i* variables start with *i* = 1 (individual variables; all possible combinations for each *i* are tested up to a maximum of 5,000,000 combinations). For all those *i* = 1 models, the delete-one-year CVSE is calculated, and the model with minimum CVSE and its associated variable are identified. Then, combinations of *i* = 2 variables are tested, and the model with minimum CVSE for *i* = 2 variables is compared to that for *i* = 1. If the *i* = 2 model has lower CVSE, its variables are identified, and the procedure continues for *i* = 3 in the same manner. Otherwise, if *i* = 1 has the lowest CVSE, the *i* = 1 model is identified as the best parsimonious model, and the procedure stops for that district. Source: adapted from a previous study [29]. Abbreviation: CVSE, cross-validation standard error.

Once the parsimonious quasi-optimal models were fitted, the same covariates from the GCM data were used in the resulting models to generate estimations of dengue for the mid-century. Maps of the difference of future (2030–2065) minus historical (1985–2015) dengue estimations were then produced. A flow chart of the procedure can be found in Fig. 1. The computed changes in total number of dengue cases were divided by the 2022 population of each district to provide an indication of incidence. An analysis included in Fig. S1 shows that the use of the 2011 census population does not significantly change the results.

## 3 Results

**Fig. 2:**
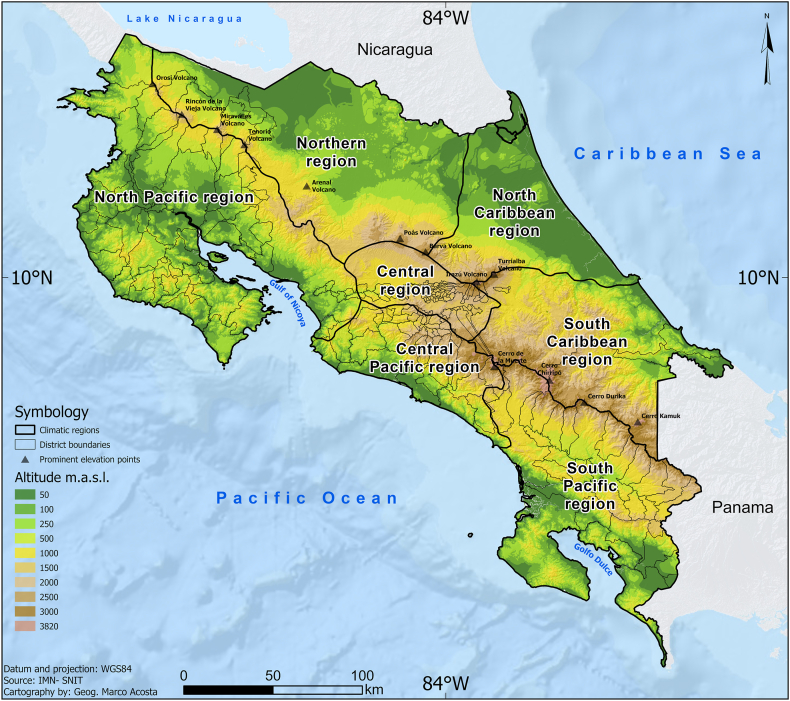
Climate regions (larger named divisions) and administrative districts (smaller divisions) of Costa Rica according to the National Meteorological Institute of Costa Rica (IMN in Spanish) and the National Geographical National Institute of Costa Rica (IGN in Spanish), respectively. Notes: Climate regions represent the largest climate divisions, while the districts represent the smallest administrative divisions. Dengue cases are reported at the district level. Data source: National Meteorological Institute of Costa Rica (IMN in Spanish) [26] and National Territorial Information System (SNIT in Spanish) [26]; map was created using ArcGis Pro software (version 3.3.1).

**Fig. 3:**
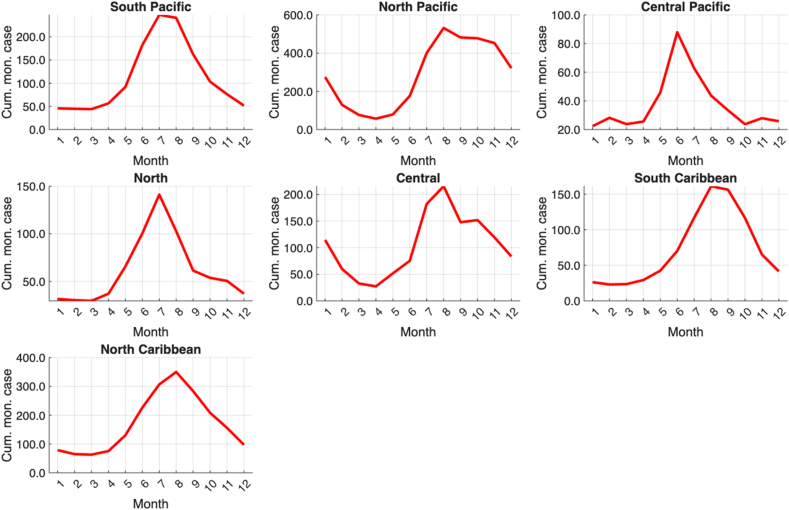
Seasonal cycle (2011–2023) of the cumulative monthly dengue cases (Cum. mon. case) reported in the districts that compose each climate region (see Fig. 2). Note: The y-axis scales differ across subgraphs. Data source: Costa Rica Ministry of Health; restricted dataset only available for this study.

**Fig. 4:**
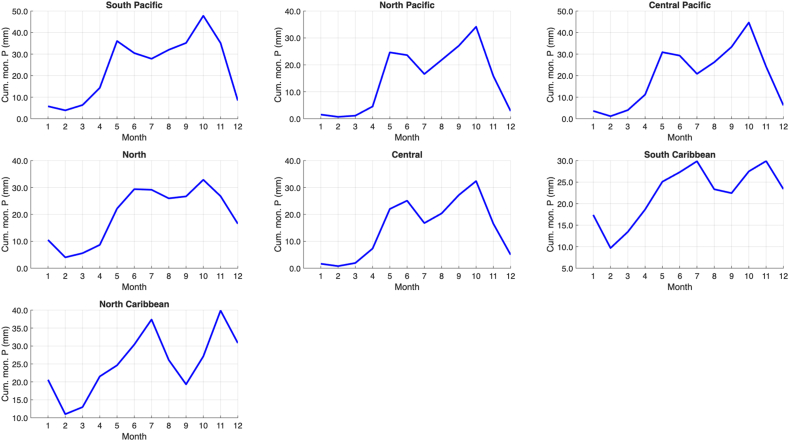
Seasonal cycle (2011–2023) of the cumulative monthly precipitation (Cum. mon. P) reported in the districts that compose each climate region (see Fig. 2). Note: The y-axis scales differ across subgraphs. Data source: Climate Hazards Group InfraRed Precipitation with Stations (CHIRPs) dataset [18].

**Fig. 5:**
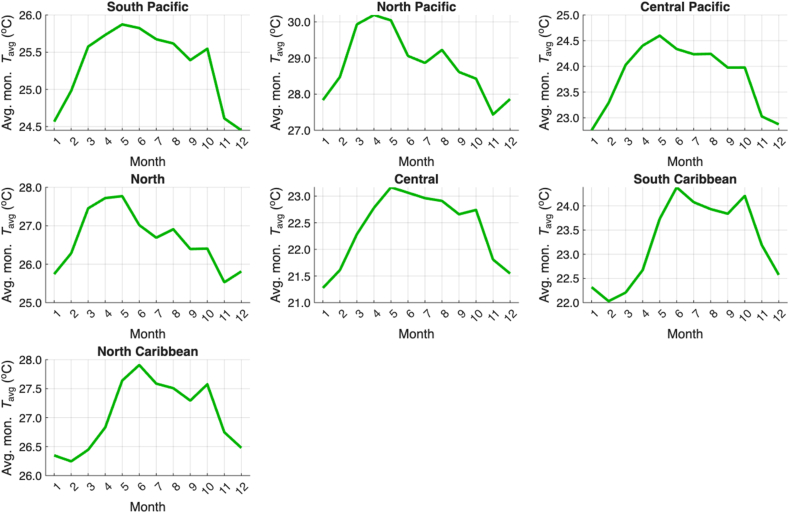
Seasonal cycle (2011–2023) of the average monthly temperature (Avg. mon. *T*_avg_) reported in the districts that compose each climate region (see Fig. 2). Note: The y-axis scales differ across subgraphs. Data source: Climate Hazards Center Infrared Temperature with Stations (CHIRTs) dataset [19].

The climate regions of Costa Rica can be found in Fig. 2. The seasonal cycles of the average monthly sums of the dengue cases of all districts in each climate region are shown in Fig. 3, and the corresponding seasonal cycles of precipitation and temperature are shown in Figs. 4 and 5, respectively. In general, dengue cases peak in July–August in most of the climatic regions; these peaks are associated with precipitation decrease during the mid-summer drought [32] in the Pacific Slope and maximum peaks in the Caribbean Slope [33]. Although in some regions the dengue peaks do not correspond to the maximum precipitation peaks of the rainy season, the possible 1–3 month lags between the climate-dengue response and the May or June precipitation peaks may also play a role [32]; a similar hypothesis can be drawn for temperature. In summary, wet and hot conditions may be associated with higher total dengue cases. A description of the main annual cycles of temperature and precipitation in Central America can be found in a study by Taylor and Alfaro [34]. Conversely, the dengue minimum in these regions is generally associated with lower temperatures and reduced precipitation. These generalizations can help verify the sign of the regression equations in the linear models to be fitted in the following sections.

Table 3 shows the top six variables (corresponding to the number of districts) most frequently appearing in the fitted models, for models calibrated for each month of the year and for the annual case. As shown, temperature variables dominate the top six predictors found in feasible regression models, except for August, September, and October (generally part of the second and largest peak of the wet season), where precipitation indices dominate. Notably, in some models, climate variables (precipitation and temperatures) are lagged, in many cases by two months, suggesting that climate conditions in previous months provide the necessary factors for dengue outbreaks in subsequent months (i.e., vector density increase and virus extrinsic and intrinsic incubation periods). However, some climate variables at lag zero also influence dengue cases in that same month. This suggests that additional processes operating at shorter timescales (e.g., weekly or daily variations) not captured in monthly data may be related to disease development and reporting delays at health centers.

**Table 3:** The top six variables (corresponding to the number of districts) most frequently appearing in the fitted models, for models calibrated for each month of the year and for the annual case.

January						
Variable	*T*_avg__l−2	*T*_min__l−1	*T*_max__l−2	days*P*_l0	*T*_min__l−2	*T*_max__l−1
Times appearing	57	53	51	50	49	48
February						
Variable	*T*_min__l−1	*T*_avg__l−1	*T*_max__l0	*T*_min__l0	days*P*_l−2	*P*_mon__l−2
Times appearing	62	59	56	50	50	50
March						
Variable	*T*_min__l−2	*T*_avg__l0	*T*_avg__l−1	*T*_min__l0	*T*_max__l−1	*T*_max__l0
Times appearing	42	40	39	38	38	33
April						
Variable	*T*_min__l−2	*T*_max__l0	days*P*_l0	*T*_max__l−2	*T*_avg__l0	*P*_mon__l0
Times appearing	40	38	37	37	36	34
May						
Variable	*T*_min__l−1	days*P*_l−2	*T*_max__l−1	*T*_min__l0	*T*_max__l0	*T*_min__l−2
Times appearing	31	29	28	24	21	21
June						
Variable	*T*_max__l0	*T*_min__l0	days*P*_l−2	*T*_avg__l0	*P*_mon__l−2	*P*_80__l−2
Times appearing	58	49	49	40	34	34
July						
Variable	days*P*_l−2	*T*_min__l0	*P*_mon__l−2	*P*_80__l−2	*T*_avg__l0	*P*_80__l0
Times appearing	95	64	60	60	39	38
August						
Variable	*P*_mon__l−1	days*P*_l−1	*P*_80__l−1	*P*_80__l−2	*P*_mon__l−2	days*P*_l−2
Times appearing	79	77	60	42	38	32
September						
Variable	days*P*_l0	*P*_80__l0	*P*_mon__l0	*P*_80__l−1	days*P*_l−1	*T*_max__l−2
Times appearing	48	42	39	37	34	34
October						
Variable	days*P*_l0	*P*_80__l−2	*T*_max__l−1	days*P*_l−2	*P*_80__l−1	*P*_mon__l−2
Times appearing	44	44	42	40	38	35
November						
Variable	*T*_max__l−2	*T*_max__l0	days*P*_l−2	*T*_min__l−2	days*P*_l0	*T*_avg__l−2
Times appearing	87	73	68	68	67	61
December						
Variable	*T*_max__l−1	days*P*_l−1	*T*_avg__l−1	*P*_mon__l−2	days*P*_l−2	*P*_80__l−1
Times appearing	75	60	50	47	40	37
Annual						
Variable	*T*_max__l−1	days*P*_l0	*T*_max__l0	*T*_min__l−1	days*P*_l−2	*T*_min__l−2
Times appearing	20	19	17	17	12	12

Note: Variable names: *T*_avg_, average temperature; *T*_min_, minimum temperature; *T*_max_, maximum temperature; days*P*, number of days when precipitation is greater than 0.1 mm; *P*_mon_, monthly mean precipitation; *P*_80_, number of days when the precipitation is greater than the 80th historical percentile. Each variable was analyzed at three month lags: 0, −1 and −2 (denoted as l0, l−1 and l−2, respectively).

**Fig. 6:**
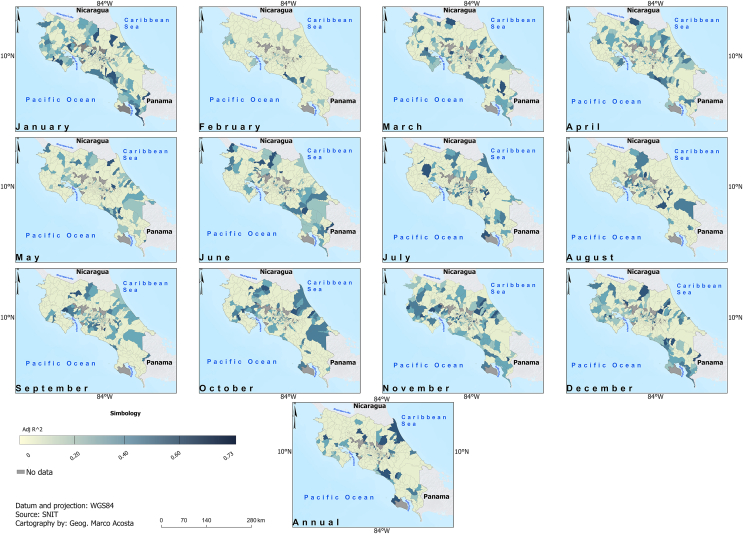
Adjusted *R*^2^ of parsimonious linear models relating climate variables to dengue total cases (2011–2023) at monthly and annual time scales. Data source: data from this study and National Territorial Information System (SNIT in Spanish) [26]; map was created using ArcGis Pro software (version 3.3.1).

**Fig. 7:**
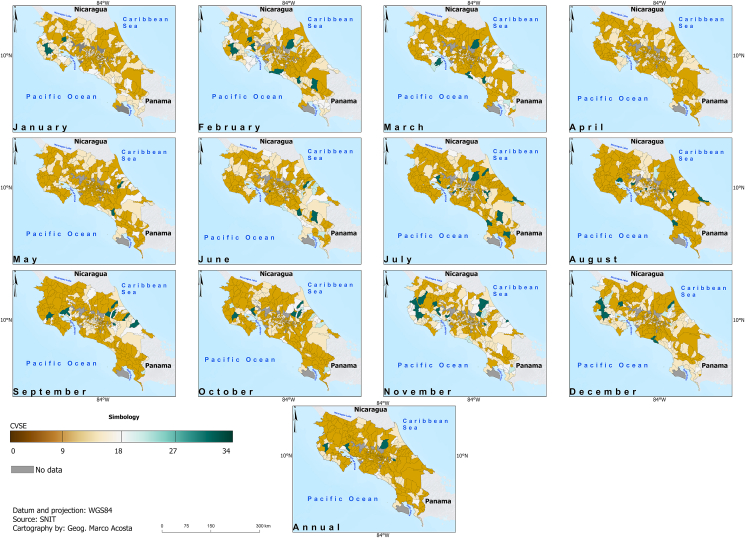
Spatial distribution of Cross-Validation Standard Error (CVSE) derived from parsimonious linear models constructed for the environmental variables through the procedure described in Fig. 1. Data source: data from this study and National Territorial Information System (SNIT in Spanish) [26]; map was created using ArcGis Pro software (version 3.3.1).

**Fig. 8:**
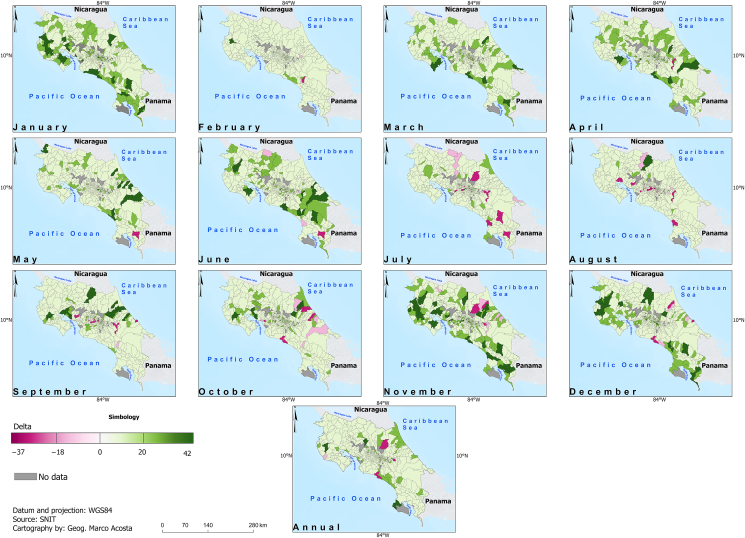
Projected changes in dengue cases between the future period (2035–2065, SSP5-8.5 scenario) and the historical period (1985–2015) at district level. Dengue cases were estimated using parsimonious linear models selected through the procedure described in Fig. 1, driven by climate projections downscaled to (1 km × 1 km) resolution and averaged to district level from our previous study [13]. Data source: data from this study and National Territorial Information System (SNIT in Spanish) [26]; map was created using ArcGis Pro software (version 3.3.1).

**Fig. 9:**
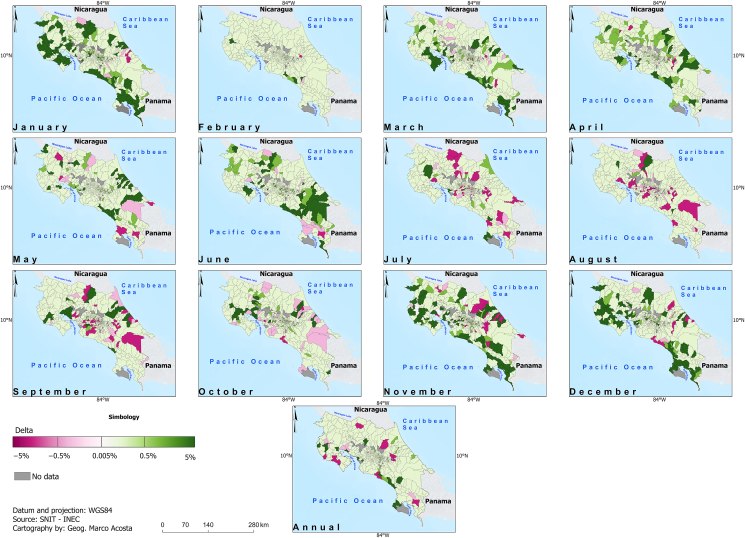
Projected changes in dengue case incidence (%) between the future period (2035–2065, SSP5-8.5 scenario) and the historical period (1985–2015) at district level. Values were calculated by normalizing the dengue case estimates (derived using parsimonious models described in Fig. 1) to the 2022 population of each district. Data source: data from this study, National Territorial Information System (SNIT in Spanish) [26], and National Institute of Statistics and Censuses (INEC in Spanish) [28]; map was created using ArcGis Pro software (version 3.3.1).

Figs. 6 and 7 display the adjusted *R*-square (Adj. *R*^2^) and the CVSE for the fitted models linking climate to dengue. While no consistent skill pattern is evident, the Pacific Slope districts demonstrate higher skill in January, whereas the Caribbean districts show higher skill in October. Interestingly, most projected future changes occur in the Pacific Slope (Figs. 8 and 9) during part of the dry season (November, December, and January). In particular, within the Pacific Slope, the North Pacific Region exhibits high seasonal dengue total cases, while other districts usually show low seasonal total cases (Fig. 3). In contrast, the highest projected increases in dengue total cases for the Caribbean Slope occur in April, May, and June, a transitional period between the seasonal minimum and the August–September peak.

## 4 Discussion

In Costa Rica, future changes in dengue total cases suggest potential increases of approximately 45 cases above the historical baseline (1985–2015) during mid-century (2035–2065) in some of the districts, under the most pessimistic concentration scenario (SSP5-8.5). While this represents a potential increase in favorable climate conditions for dengue development, other non-climatic factors are also required for outbreak development. These include the presence of infected and susceptible individuals, entry of new serotypes, travel patterns, population behavior, rapid urbanization, population growth, vector control measures, etc. The use of a pessimistic climate scenario represents, in a certain way, the most critical set of conditions for potential case development. Nevertheless, considering temperature as an important controlling factor in dengue modeling, and given the use of the most pessimistic scenario for mid-century estimates, the magnitude of projected changes is consistent with other studies [7,35].

It is important to note a limitation of the linear models used in this study to simulate the temperature–dengue relationship. Recent studies consistently show that temperature is a key, but nonlinear, driver of dengue total cases. A global meta-analysis found the temperature–dengue relationship peaks near 24 °C and weakens at cooler and hotter temperatures, with effects further shaped by rainfall and socioeconomic context, implying that warming can strengthen transmission primarily in regions where climates sit near that optimum and where variability is high [7]. Complementing this, a 2023 systematic review (54 studies analyzed) estimated that each 1 °C increase in high temperatures raises dengue risk by approximately 13%, with larger effects in tropical monsoon and humid subtropical zones [6]. Many districts of Costa Rica experience the temperature conditions described in these studies, which justifies the projected sharp increases in dengue total cases. However, uncertainty in multiple variables, such as those associated with non-linearities climate model projections, concentration scenario, dengue variables and similar biological aspects, and previous exposure of the population to the different dengue serotypes, limits the predictive precision. Therefore, the solution presented here should be interpreted as only a guide to identify areas potentially at risk for increased dengue transmission rather than as definitive forecasts. These results may help authorities prioritize surveillance efforts and inform continued study of dengue and other vector-borne diseases dynamics. For this reason, projections were not extended far beyond 2065, when uncertainty is much greater. This work aims to guide adaptative measures that need continuous refinement as new evidence emerges [36].

Without excluding the influence of temperature, precipitation is a dominant factor during July, August, September, and October, months of high precipitation on both slopes of Costa Rica. Recent studies show that precipitation influences dengue in context-dependent, lagged, and often nonlinear ways. A 2022 systematic review and meta-analysis reported that monthly precipitation shows the strongest pooled correlation with dengue among common climate variables, typically peaking at 1–3-month lags (*e.g*., *r* ≈ 0.38), consistent with rainfall creating breeding sites for *Aedes* mosquitoes [8]. The same authors highlight a dual role: in the Philippines and Puerto Rico, rainfall increased dengue where dry-season length varies greatly (more intermittent rains and containers), but decreased dengue where dry seasons are more regular, likely due to “flushing” of larval habitats during heavy, sustained rains. Consistent with these findings, in our case, dengue outbreaks lag precipitation variables at 0, −1, and −2 months, with −2 months lags frequently observed. It is interesting to note that the relationships at lag 0 with precipitation and temperature variables suggest that small lags may reflect dengue responsiveness to climate occurring at a sub-monthly scale, which cannot be resolved by the monthly resolution of the data.

Communities can reduce climate change–amplified dengue risk by adopting integrated vector management that prioritizes environmental control (routine removal of container habitats, reliable solid-waste services, and secure, covered household water storage) alongside other targeted larval source management (*e.g*., *Bacillus thuringiensis israelensis,* temephos or pyriproxyfen where appropriate) and adult chemical control guided by entomological surveillance and insecticide-resistance monitoring. Climate-informed early-warning systems that couple seasonal forecasts with local case and vector data should trigger pre-emptive, neighborhood-level actions before high-risk heat–rainfall windows. Urban adaptation, expanded piped water, covered tanks, improved drainage and stormwater management to prevent standing water, and housing measures such as window/door screens confer co-benefits for other climate hazards. Sustained, equity-focused community engagement (schools, workplaces, and faith groups), clear risk of communication, and provision of low-cost protective materials (lids, screens, and repellents) strengthen adherence. Finally, coordinated governance that aligns municipal sanitation, public health, and climate services, with routine monitoring and evaluation, underpins durable, climate-resilient dengue prevention.

These climate-driven projections of higher mid-century dengue suitability under SSP5-8.5 have immediate implications for integrated urban, environmental, and public-health planning, because realized transmission will be co-produced by climate variability and modifiable features of the built environment, infrastructure, and vulnerability [37,38]. Urban densification and informal growth can elevate exposure via poor housing protection, urban heat, unreliable piped water that increases household storage, and deficits in drainage and solid-waste services that sustain container habitats [[39], [40], [41], [42]]. This is consistent with lagged and nonlinear precipitation effects and compound climate extremes shaping outbreak risk [43]. Environmental planning should therefore prioritize climate-resilient stormwater and drainage design and maintenance, water-security interventions (continuous supply and safe storage standards) that are assessed for unintended vector consequences, and heat-mitigation measures that avoid inadvertently creating water-holding features [39,41]. Public-health systems can translate climate information into decision triggers by coupling seasonal forecasts with local surveillance and entomological data to deploy pre-emptive, neighborhood-scale source reduction, targeted larval control, resistance-aware adult control, and surge capacity in clinical readiness, while improving temporal resolution to capture sub monthly dynamics [[43], [44], [45]]. Finally, an equity-centered governance framework is essential: investments should prioritize districts where climatic suitability intersects with service deficits and poverty, and performance should be evaluated not only by case reduction but also by feasibility, distributional impacts, and co-benefits for flood, heat, and water-related risks [42,46].

Building on Hidalgo's [36] emphasis that rising temperatures and shifting hydroclimate variability can increase aridity and strain water systems (with knock-on effects for exposure and water-related services), and on Ley et al. [3] who warn that Central America's climate change adaptation gap is growing because of vulnerability, uneven capacity, and limited learning from adaptation projects persist, a next-generation climate–society coupled model aimed at reducing vector-borne disease impacts should explicitly link (1) downscaled climate–hydrology hazards (temperature, rainfall extremes, drought indices/El Niño-Southern Oscillation (ENSO)-relevant variability, as well as water availability and intermittency) to (2) social and health-system state variables that govern exposure and response (urbanization rate and informal-settlement growth, access to piped water and household storage practices, sanitation and drainage coverage, land-use change, human mobility, poverty/inequality, vector-control workforce and insecticide coverage, and medical expenditure and primary-care access as proxies for detection and treatment capacity), while also representing feedbacks in which water-management adaptations (e.g., storage expansion, rationing schedules, irrigation, stormwater infrastructure) can unintentionally create or remove breeding sites. To be decision-relevant, the model should be calibrated with routine surveillance and climate/streamflow observations, tested under co-produced adaptation scenarios (targeted investments in water reliability, drainage, and health spending; early warning triggers; and prioritization rules during drought), and evaluated not only on epidemiological fit but also on equity and implementation metrics (who benefits, and where the gap remains), enabling water managers and health authorities to identify “no-regrets” interventions that reduce climate-sensitive transmission risk while shrinking the adaptation gap highlighted for the region. Future work will explore the comparison of this methodology to non-linear methods [47] to determine whether this methodology influences non-linearities in the results.

## 5 Conclusions

Temperature and precipitation data are significantly related to district-level dengue counts in Costa Rica, where temperature is most relevant in the dry season and precipitation in the rainy season. Moreover, mid-century projections of dengue cases show increments of up to 42 more cases in some districts compared to the historical scenario, but the areas with the greatest changes vary by month. Although the distribution and incidence of dengue cases also depend on non-climate factors, results based on the projected climate change of the most pessimistic scenario represent a valuable guide of where dengue incidence increases may appear in the future. These climate-driven projections of dengue suitability have immediate implications for integrated urban, environmental, and public-health planning and should be part of a continuous adaptive effort to guide health policies aimed at reducing the impacts of climate change on health in areas with high dengue risk.

## CRediT authorship contribution statement

**Hugo G. Hidalgo:** Writing – review & editing, Writing – original draft, Visualization, Validation, Supervision, Resources, Project administration, Methodology, Investigation, Funding acquisition, Formal analysis, Conceptualization. **Eric J. Alfaro:** Writing – review & editing, Writing – original draft, Validation, Supervision, Resources, Investigation, Conceptualization. **Fabio Sanchez:** Writing – review & editing, Writing – original draft, Validation, Supervision, Resources, Methodology, Investigation, Conceptualization. **Adriana Troyo:** Writing – review & editing, Validation, Resources, Investigation, Formal analysis, Data curation, Conceptualization. **Tito Maldonado:** Writing – review & editing, Software, Resources, Investigation, Formal analysis, Data curation. **Zaray Miranda-Chacón:** Writing – review & editing, Resources, Methodology, Investigation. **Eric Morales-Mora:** Writing – review & editing, Supervision. **Monserrat Solano-Gamboa:** Writing – review & editing, Resources, Investigation, Data curation. **Marco Acosta-Quesada:** Software, Resources, Formal analysis, Data curation.

## Declaration of generative AI and AI-assisted technologies in the manuscript preparation process

During the preparation of this work, the author(s) used ChatGPT v5.2 in order to reorganize the introduction following one reviewer's request. After using this tool/service, the authors reviewed and edited the content as needed and take full responsibility.

## Funding

The authors declare that financial support was received for the research and/or publication of this article. This study was funded by the 10.13039/100000002National Institutes of Health 10.13039/100000061Fogarty International Center (grant number D43TW011403) for the project entitled “International Training Program in Environmental Health over the Lifespan” (Claudio L and van Wendel de Joode B, PIs), a grant awarded to the 10.13039/100007277Icahn School of Medicine at Mount Sinai and Universidad Nacional, Costa Rica (SIA 0019-23 EcoSalud). Additional support was provided by Vicerrectoría de Investigación, 10.13039/501100005298Universidad de Costa Rica grant: Influencia del cambio climático en la incidencia de enfermedades tropicales infecciosas: El caso de Cuajiniquil, La Cruz, Guanacaste, Costa Rica (EcoSalud, C4226). HH and EA wish to also acknowledge the UCR projects C6459, C5279, C5067, C2103, C3991 (UCREA), A4906 (PESCTMA), C3195 and B0-810. EA, and HH were partially supported by a grant awarded by the 10.13039/501100000193International Development Research Centre (10.13039/501100000193IDRC), Ottawa, Canada, and the Central American University Council (CSUCA-SICA) to the Red Centroamericana de Ciencias sobre Cambio Climático (RC4) project (CR-66, C4468, SIA 0054-23, the opinions expressed here do not necessarily represent those of IDRC, CSUCA, or the Board of Governors).

## Conflict of interest

We declare no conflicts of interest.
