# Supplementary material for: High-resolution climate–dengue modeling and mid-century projections under SSP5-8.5 in Costa Rica
Source: Sci One Health. 2026 Apr 27;5:100157. doi: 10.1016/j.soh.2026.100157 (PMC13223965; doi:10.1016/j.soh.2026.100157)
Supplement: Multimedia component 1 [file mmc1.docx]

**Supplementary material**


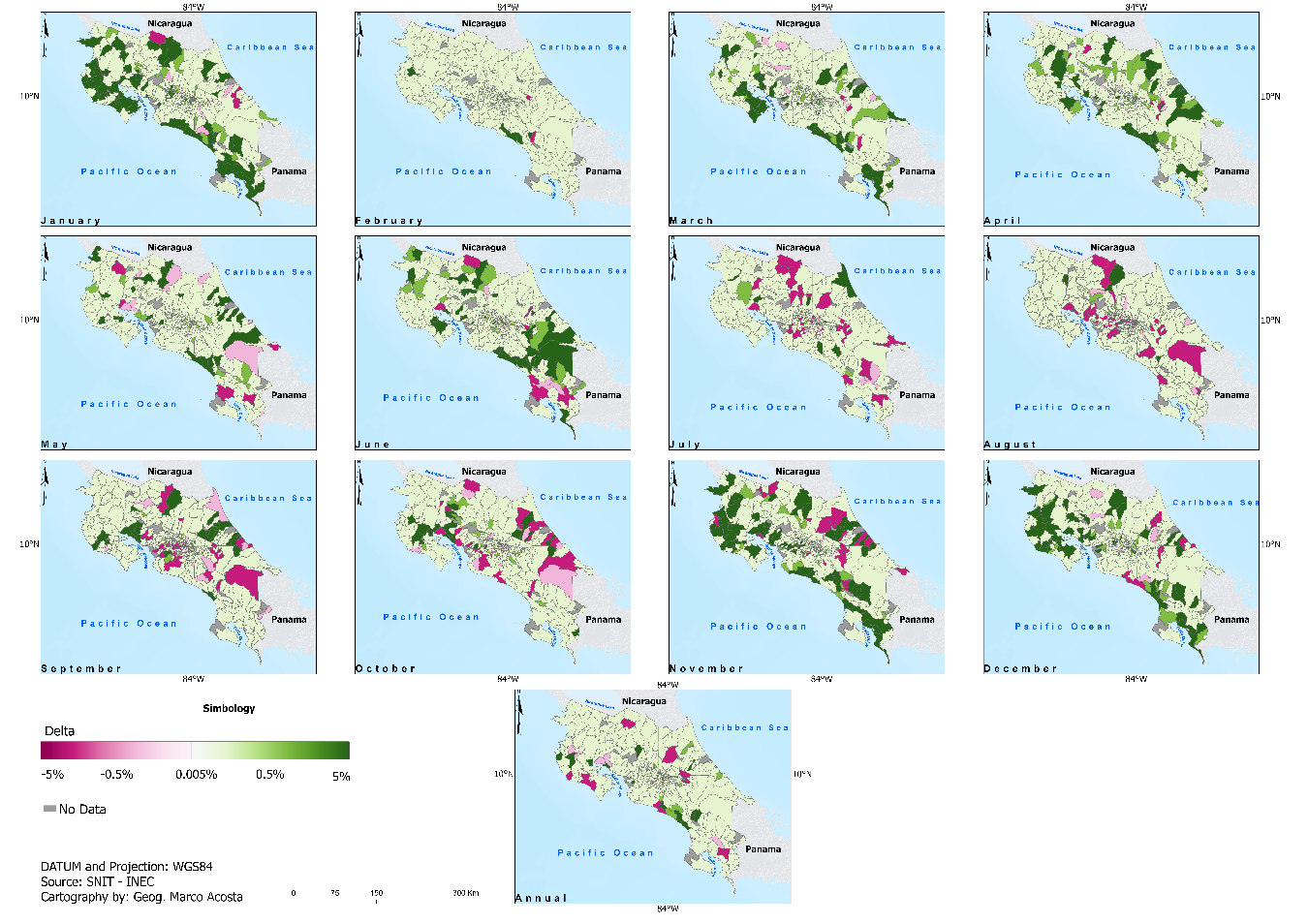


**Fig. S1.** Future (2035–2065) vs. historical scenario (1985–2015) in dengue counts divided by the 2011 population of each district (%) to account for incidence. In Fig. S1, the equivalent of Fig. 9 using the 2011 population is shown. The change in incidence is not significant and the model solutions are not sensitive to the choice of population census. Data source: data from this study, National Territorial Information System (SNIT in Spanish) [27], and National Institute of Statistics and Censuses (INEC in Spanish) [29]; map was created using ArcGis Pro software (version 3.3.1).
